# GenAI Literacy and Students’ Exploitative and Exploratory Innovation: The Moderating Role of Critical Thinking

**DOI:** 10.3390/jintelligence14100241

**Published:** 2026-10-02

**Authors:** Yali Tang, Liang Wu

**Affiliations:** 1 Jewelry Institute, Guangzhou Polytechnic University, No. 1342 Shi Liang Road, Guangzhou 511483, China; tyl0107@126.com; 2 College of Education for the Future, Beijing Normal University, No. 18 Jin Feng Road, Zhuhai 519087, China; 3 Center for Cantonese Merchants Research & Institute of Studies for the Greater Bay Area, Guangdong University of Foreign Studies, No. 178 Waihuan Dong Road, Guangzhou 510006, China

**Keywords:** GenAI literacy, exploitative innovation, exploratory innovation, critical thinking, college students

## Abstract

Generative artificial intelligence (GenAI) literacy, encompassing the knowledge, skills, and attitudes for effective use, is becoming essential in education. However, its distinct associations with exploitative versus exploratory innovation and the role of critical thinking remain unclear. Grounded in social cognitive theory, this study surveyed 228 students to examine these relationships. GenAI literacy was positively associated with exploitative innovation and showed a U-shaped association with exploratory innovation, in which benefits emerged only after a literacy threshold; the U-shaped association was robust to correction for multiple comparisons. Critical thinking negatively moderated the association between GenAI literacy and exploitative innovation, and this interaction was robust to correction for multiple comparisons; the hypothesized moderation of the U-shaped exploratory association, in contrast, did not reach the 0.05 level and did not receive empirical support. The findings extend theory by differentiating how GenAI literacy relates to distinct innovation types and offer practical guidance—drawn from the supported associations and from theoretical reasoning—for fostering balanced human–AI collaboration. Because participation was voluntary, the self-selected sample may have overestimated the reported associations, and all results should be interpreted with this caveat in mind.

## 1. Introduction

The rapid and pervasive adoption of generative artificial intelligence (GenAI) is fundamentally reshaping the landscape of learning, creativity, and innovation (Cloux et al., 2025; Ji et al., 2025; Krakowski, 2025). Within educational settings, this transition calls on students to develop a new competence—GenAI literacy—defined as the knowledge, skills, and attitudes that enable individuals to engage with, make sense of, and capitalize on generative AI tools (Daniel et al., 2025; García-López et al., 2025; Zhang et al., 2025). As students increasingly engage in human–machine co-creation, a pressing question emerges: How does the development of GenAI literacy influence students’ capacity for innovation?

Scholarly interest in the innovation outcomes of technology adoption is well-established, often framed through the lens of organizational ambidexterity, which distinguishes between exploitative innovation (incremental refinements and efficiency improvements) and exploratory innovation (radical search and experimentation) (He & Wong, 2004; March, 1991). Recent evidence on trust in GenAI points to a more nuanced landscape: it appears to facilitate exploitative innovation in a straightforward, positive manner, while its association with exploratory innovation follows an inverted U-shaped pattern (Lin et al., 2025). Even so, the mechanisms underlying this asymmetry—and their implications for fostering both innovation forms—remain largely unaddressed. Existing studies primarily focus on organizational or employee contexts, leaving the direct effects of students’ foundational GenAI literacy on their ambidextrous innovative behaviors underexplored. Furthermore, potential negative impacts (e.g., idea homogenization, over-reliance) and critical boundary conditions are seldom examined in student populations (Yang et al., 2025).

To address these gaps, we adopt Social Cognitive Theory (SCT) (Bandura, 1986) as our overarching framework. SCT posits a triadic reciprocal determinism between personal factors, behaviors, and the environment. Within this view, GenAI literacy is a key personal competency that differentially impacts innovation types. Specifically, we theorize that GenAI literacy supports exploitative innovation through a positive linear pathway by enhancing efficiency and self-efficacy in refining tasks (Shi et al., 2025). In contrast, its relationship with exploratory innovation is hypothesized as U-shaped: at low-to-moderate levels, overdependence or superficial use may hinder novel ideation (Guo et al., 2025); only advanced literacy, enabling critical use of AI as a “cognitive collaborator,” unlocks its full exploratory potential (Park, 2026).

Moreover, critical thinking—conceptualized as the capacity to scrutinize, appraise, and integrate information (T. Li et al., 2024)—is posited to act as an important cognitive boundary condition. As GenAI tools become more accessible, a key risk is that proficiency may not translate into effective and critical application (Zhang et al., 2025). From an SCT perspective, critical thinking acts as a cognitive self-regulatory mechanism (Y. Zhao et al., 2025). Accordingly, we argue that this competence attenuates the positive association between GenAI literacy and exploitative innovation, insofar as it curbs excessive dependence on GenAI outputs; conversely, it sharpens the U-shaped association with exploratory innovation, as students who think critically are better positioned to appraise and assimilate novel AI-generated ideas.

**Figure 1:**
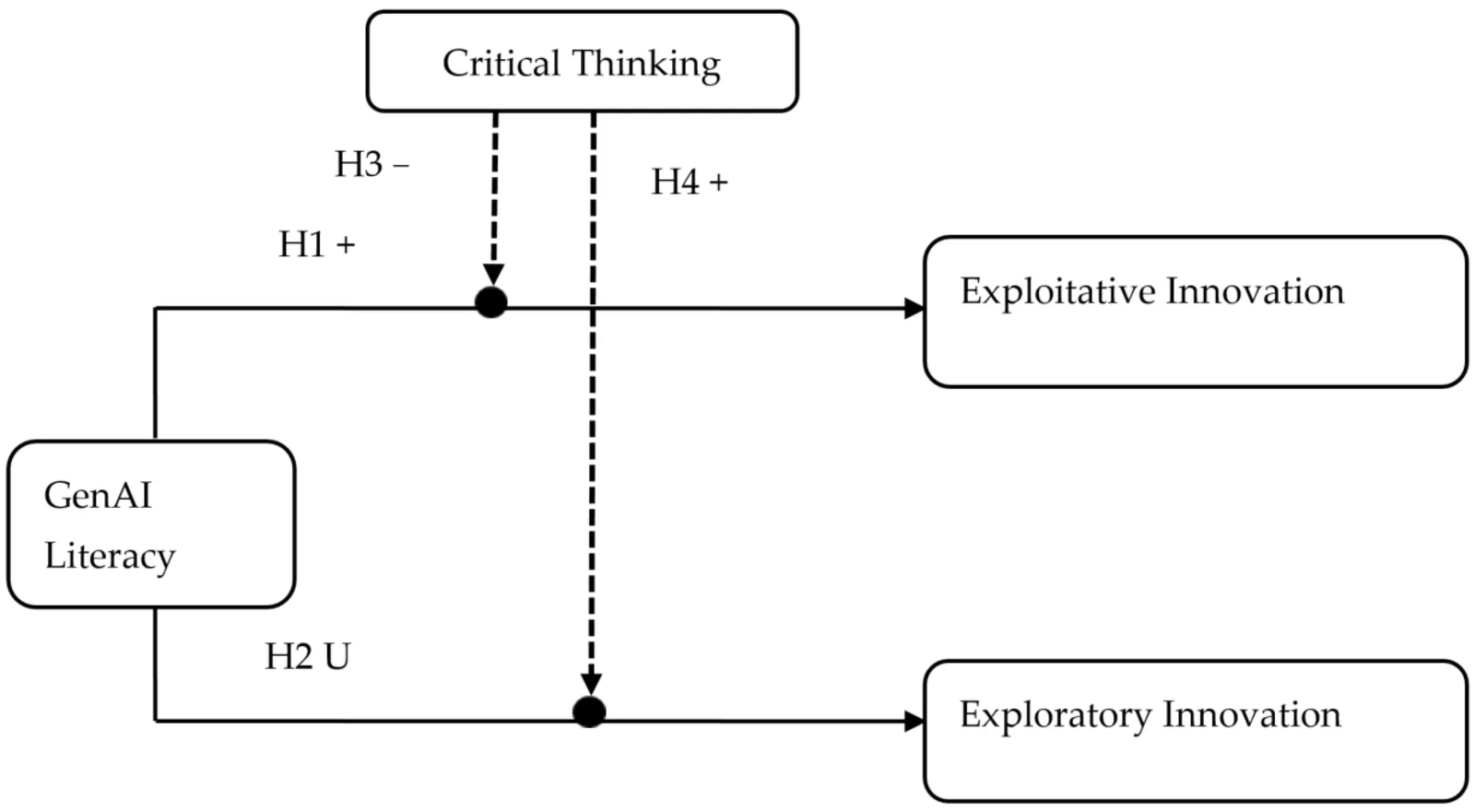
Proposed conceptual model of the relationships among GenAI literacy, critical thinking, and students’ exploitative and exploratory innovation. Note. Solid arrows denote hypothesized direct effects; dashed arrows denote moderating effects. H1: GenAI literacy positively relates to exploitative innovation (+). H2: GenAI literacy exhibits a U-shaped relationship with exploratory innovation (U). H3: Critical thinking negatively moderates the relationship between GenAI literacy and exploitative innovation (−). H4: Critical thinking positively moderates the U-shaped relationship between GenAI literacy and exploratory innovation. + = hypothesized positive relationship; − = hypothesized negative relationship; U = hypothesized U-shaped (curvilinear) relationship.

Taken together, this research responds to the lacunae identified above by examining how GAI literacy shapes students’ exploitative and exploratory innovation, with critical thinking positioned as a central boundary condition. Grounded in SCT, this research makes several contributions. First, it advances theory by distinctly modeling heterogeneous effects (linear vs. U-shaped) of GenAI literacy on innovation types. Second, it theorizes critical thinking as a dual-role boundary condition and documents a robust negative moderation of the GenAI literacy–exploitative innovation link, while treating the hypothesized amplification of the U-shaped exploratory association as an open empirical question. Third, it extends non-linear innovation models from organizational research to the distinct educational context. Figure 1 displays the conceptual model.

## 2. Theory and Hypotheses

### 2.1. GenAI, Exploitative and Exploratory Innovation

Rooted in March’s (1991) seminal work, the concepts of exploration and exploitation are central to organizational learning and innovation. In innovation research, exploitative innovation focuses on refining existing knowledge, while exploratory innovation aims for breakthrough solutions (He & Wong, 2004; Jansen et al., 2006). This dichotomy also applies to student learning contexts (Ding et al., 2023).

Research on GenAI’s impact on these innovation types shows mixed results. Some studies highlight its positive role in enabling both forms by generating ideas and optimizing processes (Singh et al., 2024; Wael AL-khatib, 2023; Wang et al., 2025). Others suggest contingent effects, moderated by factors like environmental dynamism, ethical concerns, or usage patterns (Khan et al., 2025; Singh et al., 2024; Zhang et al., 2025). At the individual level, studies indicate that work-related GenAI use promotes exploitative learning, while non-work-related use fosters exploratory learning (Zhang et al., 2025).

While providing a theoretical foundation, existing research primarily focuses on organizational or employee contexts, leaving the direct effects of GenAI literacy on student innovation underexplored.

### 2.2. Unsolved Questions: GenAI Literacy’s Impact on Student Innovation

While research shows GenAI literacy enhances student outcomes like creativity and academic performance (Alzubi et al., 2025; Ji et al., 2025), key questions remain regarding its specific impact on exploitative versus exploratory innovation.

First, the differential effects are unclear. Existing studies often treat innovation generically, failing to test how GenAI literacy distinctly influences these two types (Y. Hwang & Wu, 2025; Ji et al., 2025).

Second, potential negative impacts are neglected. Research in other contexts warns of risks like idea homogenization and diminished intrinsic motivation (Krakowski, 2025; Sabbah & Li, 2025), but these are seldom examined in student innovation.

Third, boundary conditions remain under-explored. While organizational research has highlighted contextual moderators such as environmental dynamism (L. Li et al., 2025), and individual-level studies have identified factors such as cognitive style (Sun et al., 2025), little research has explicitly investigated moderators of the relationship between GenAI literacy and innovation in student populations—a gap this study addresses by examining critical thinking as a moderator.

### 2.3. The Effects of GenAI Literacy on Students’ Exploitative and Exploratory Innovation

Based on SCT, this study posits that students’ GenAI literacy—a key personal factor—interacts with the AI environment to shape innovative behaviors (Bandura, 1986; Shahzad et al., 2025). We hypothesize that GenAI literacy positively facilitates exploitative innovation, which focuses on refining existing knowledge for incremental gains (He & Wong, 2004; March, 1991). This effect is driven by three SCT-based mechanisms:

First, it fosters a cyclical reinforcement of mastery experience and self-efficacy. Successful use of GenAI for optimization tasks provides enactive mastery, strengthening students’ belief in their ability to leverage AI (Shahzad et al., 2025). This heightened self-efficacy increases their effort and persistence in exploitative activities, creating a virtuous cycle that drives incremental improvement (Bandura, 1989; Zhang et al., 2025).

Second, GenAI serves as a model for observational learning. Students with high literacy can observe and internalize the efficient, rule-based optimization processes demonstrated by AI systems (Ji et al., 2025). By learning these methods and standards, they can more systematically use AI as a tool to exploit and refine their existing knowledge base (Bandura, 1999; Wang et al., 2025).

Third, the process exemplifies triadic reciprocal causation. GenAI literacy (personal factor) enables students to proactively shape their use of the AI environment (behavior), which in turn provides feedback and efficiency gains that reinforce their efficacy and sustained engagement in exploitative innovation (Bandura, 2001). This dynamic, mutually shaping interaction systematically promotes exploitative innovation (Ji et al., 2025). Therefore,

**H1.**  *GenAI literacy is positively correlated with students’ exploitative innovation.*

Exploratory innovation fundamentally involves departing from established knowledge to experiment with new ideas and domains (March, 1991). Grounded in SCT, this study hypothesizes that GenAI literacy influences such innovation through a U-shaped relationship. The facilitative effect is modest at low-to-moderate competency levels but accelerates significantly once literacy surpasses a critical threshold. This curvilinear dynamic is explained by three SCT-based mechanisms.

First, the non-linear growth of resilient creative self-efficacy through mastery. While performance accomplishments build self-efficacy (Bandura, 1989), resilient self-efficacy for uncertain exploration requires mastering difficulties through sustained effort (Bandura, 1989). At low-to-moderate literacy, students experience fragmented successes with high trial-and-error costs, resulting in weak, vulnerable efficacy (Ji et al., 2025). High literacy enables sustained mastery of complex exploratory tasks (e.g., iterative prompt optimization for novel ideation). This forges strong, resilient creative self-efficacy (Bandura, 2001; Wang et al., 2025), which in turn fuels greater effort and persistence, creating a self-reinforcing cycle that explains the accelerating effect post-threshold (Ji et al., 2025).

Second, the threshold for abstract modeling and generative rule extraction. Observational learning at its highest level involves abstract modeling—extracting the underlying rules governing diverse behaviors (Bandura, 1999). High GenAI literacy enables students to move beyond mimicking specific AI outputs (Wang et al., 2025). They can extract the underlying ‘styles of thinking’ and generative rules the AI employs for novel search and combination (Krakowski, 2025). Internalizing these rules as cognitive guides allows for active, rule-based synthesis and the generation of truly novel behavior (Krakowski, 2025), representing a qualitative leap in exploratory capability that requires a competency threshold (Ji et al., 2025; L. Zhao et al., 2025).

Third, triadic reciprocal causation enables agentic shaping of the exploratory environment. SCT posits that personal, behavioral, and environmental factors interact bidirectionally (Bandura, 2001). High GenAI literacy transforms this interaction: Students can proactively shape the AI environment through sophisticated exploratory dialogues (behavior). This transforms AI into a dynamic “virtual partner” (Wang et al., 2025) whose novel responses (environment) stimulate new cognitive connections (personal factor). This deep, synergistic co-evolution between user agency and AI’s generative potential is essential for breakthrough exploration and is contingent on high literacy (Lin et al., 2025). At lower levels, the interaction is more reactive, limiting synergistic potential (Ji et al., 2025). Therefore,

**H2.**  *GenAI literacy has a U-shaped relationship with students’ exploratory innovation, such that the positive effect strengthens at higher levels of literacy.*

### 2.4. Moderating Effects of Critical Thinking

Building on the SCT framework (Bandura, 2001), this study proposes that an individual’s critical thinking serves as a crucial boundary condition (Ji et al., 2025; Lin et al., 2025), negatively moderating the positive relationship between GenAI Literacy and exploitative innovation (List & Sun, 2023; Lombardi, 2023). As a core self-regulatory mechanism (Bandura, 1991), critical thinking alters how personal competency translates into behavior through three key processes:

First, it promotes intentional self-regulation, countering automation and cognitive entrenchment. While GenAI literacy facilitates efficiency through mastery and observation, it risks fostering over-reliance on standardized AI outputs (Ji et al., 2025). High critical thinking triggers active self-monitoring and regulatory judgment during AI-assisted tasks (Chou et al., 2019), prompting students to question AI’s reasoning rather than passively accept its suggestions. This critical engagement disrupts automatic, efficiency-driven application, diverting cognitive resources from pure exploitation and thus weakening the direct literacy-to-output link (List & Sun, 2023).

Second, it alters vicarious learning and behavioral evaluation standards. Students with high literacy learn optimization standards from AI models (Wang et al., 2025). However, those with high critical thinking apply more robust, independent internal standards. They evaluate AI proposals against broader criteria like originality and ethics (Held et al., 2025; Toma & Yánez-Pérez, 2025), rather than uncritically adopting AI’s efficiency-centric logic. This cognitive filtering makes them more selective and less uniformly efficient in applying GenAI for purely exploitative purposes, consistent with findings that reduced cognitive engagement can attenuate GenAI’s positive effects (Byrnes & Dunbar, 2014; Zhang et al., 2025).

Third, it introduces a protective mechanism against path dependence in the triadic reciprocal system. High GenAI literacy can create a self-reinforcing cycle favoring exploitation (Zhou et al., 2025). Critical thinking acts as a disruptive personal factor within this system, fostering cognitive flexibility and a prevention-focused mindset against over-dependence on familiar paths (Lombardi, 2023). By generating self-reactive influences (e.g., motivation for novelty) that counter the self-reinforcing loop of exploitation (Ji et al., 2025), critical thinking weakens the link between the enabling tool (GenAI literacy) and the path-dependent behavior (exploitative innovation). Therefore,

**H3.**  *The level of critical thinking negatively moderates the relationship between GenAI literacy and students’ exploitative innovation.*

Grounded in SCT (Bandura, 1991), we further hypothesize that critical thinking exerts a positive moderating influence on the U-shaped linkage between GenAI literacy and students’ exploratory innovation (Lin et al., 2025). As a self-regulatory cognitive process (Bandura, 1991), critical thinking enhances the beneficial effects and mitigates the risks associated with GenAI use for exploration through three key mechanisms:

First, critical thinking extends the positive slope of the U-curve by deepening the “GenAI empowerment” effect. High GenAI literacy provides access to novel information (Krakowski, 2025), while critical thinking enables students to interrogate, deconstruct, and synthesize these outputs through analysis and evaluation (Facione, 1990; Liu et al., 2025) more effectively. This allows them to better leverage AI as a “virtual partner” for novel problem-solving (Wang et al., 2025), amplifying the positive effect of literacy and potentially shifting the curve’s turning point upward, akin to the moderating role of “human–AI fit” (Lin et al., 2025; Murphy et al., 2023).

Second, critical thinking elevates the trough of the U-curve by mitigating the “GenAI limitation” mechanism. The initial decline in the U-shape is linked to over-reliance and “metacognitive laziness” (Fan et al., 2025), which narrows cognitive search. Critical thinking acts as a cognitive safeguard through metacognitive monitoring and evaluative scrutiny (G. J. Hwang et al., 2025), helping students maintain agency and independent judgment. By reducing uncritical acceptance of AI outputs, critical thinking lessens the performance dip, thereby raising the lowest point of the curve (T. Li et al., 2024; Loyens et al., 2023).

Third, critical thinking flattens the descending slope by enabling reflective self-regulation and strategic adaptation. At high literacy levels, critical thinking provides the capacity for strategic foresight—a core component of human agency (Bandura, 1989). Through self-reflection, students can recognize limitations in their AI-aided strategy and consciously adapt their goals and interactions (Min et al., 2025). This shift from passive information-seeking to an iterative, review-focused exchange mitigates stagnation and fosters sustained, adaptive exploration (List & Sun, 2023). Therefore,

**H4.**  *The level of critical thinking positively moderates the U-shaped relationship between GenAI literacy and students’ exploratory innovation.*

## 3. Methodology

### 3.1. Participants and Procedure

Data were collected via a two-wave questionnaire survey from students at a university in Guangdong Province, China, in 2025. Students at this institution were considered suitable participants because of their immersion in a digital information environment and active engagement with GenAI. A back-translation procedure was employed to ensure translation equivalence. With the assistance of Ph.D. students in organizational behavior, questionnaires were distributed to voluntary participants via email or Wjx.cn (a widely used Chinese online survey platform) in two waves approximately one week apart. A total of 1500 questionnaires were distributed in Wave 1, of which 570 were returned and 526 were valid. In Wave 2, 510 of the 526 valid respondents completed the follow-up survey. After excluding 282 responses due to excessive missing data (83), failed attention checks (97), and unmatched responses (102) across the two waves, 228 matched responses were retained for analysis, yielding an effective response rate of 15.20%. Wave 1 measured control variables, GenAI literacy, and critical thinking; Wave 2 measured exploitative and exploratory innovation. Of these students, 176 (77.193%) were female, and 6 (2.632%) students were aged 18–21, 204 (89.474%) students were aged 22–26, and 18 (7.895%) students were aged above 26.

To rule out the possibility that non-response bias influenced the results, we compared the profile of early and late responders. Based on the order in which the second-wave questionnaire was returned, the final sample was split into an earlier-response subgroup (N = 108) and a later-response subgroup (N = 120). Independent-samples t-tests revealed no statistically significant differences between the two subgroups in gender (ΔM = 0.029, t(226) = 0.514, 95% CI [−0.081, 0.139]), age (ΔM = −0.039, t(226) = −0.858, 95% CI [−0.128, 0.050]), or major (ΔM = −0.007, t(226) = −0.030, 95% CI [−0.493, 0.478]). Likewise, the two groups did not differ significantly on the focal outcome variables, exploitative innovation (ΔM = −0.104, t(226) = −0.935, 95% CI [−0.323, 0.115]) and exploratory innovation (ΔM = 0.059, t(226) = 0.485, 95% CI [−0.180, 0.297]). These results indicate that the timing of survey completion was not associated with systematic variation in respondents’ characteristics or key study variables, and non-response bias is therefore unlikely to be a serious concern in this study. We note, however, that this analysis addresses non-response bias specifically; it cannot rule out self-selection bias arising from the voluntary nature of participation, a possibility we acknowledge at the end of the Results section and in Section 5.3.

### 3.2. Measures

All items listed in Table 1 were rated on a seven-point response scale, with the attitudinal items anchored at “strongly disagree” (1) and “strongly agree” (7), and the remaining items anchored at “very low” (1) and “very high” (7). Five items adapted from Liu et al. (2025) were used to measure GenAI literacy (Alpha = 0.915, composite reliability [CR] = 0.916, AVE = 0.686). The response format was adapted to a seven-point scale ranging from 1 (strongly disagree) to 7 (strongly agree) to maintain consistency with the other study measures. These items were drawn from the behaviour literacy subscale of the GenAI literacy in digital multimodal composing scale, assessing respondents’ behavioural commitment to GenAI learning and application (i.e., behavioural engagement and behavioural intention). As shown in Table 1, the measure exhibited satisfactory convergent and discriminant validity in the present sample (AVE = 0.686 > MSV = 0.183, ASV = 0.107). Exploitative innovation was gauged through three items adapted from Ding et al. (2023) and Meng et al. (2024), with reliability and convergent validity of Alpha = 0.817, CR = 0.829, and AVE = 0.622. Three further items drawn from the same sources (Ding et al., 2023; Meng et al., 2024) captured exploratory innovation (Alpha = 0.850, CR = 0.848, AVE = 0.651). Five items adapted from T. Li et al. (2024) measured critical thinking (Alpha= 0.922, CR = 0.925, and AVE = 0.715).

Following prior research (Ding et al., 2023; Ji et al., 2025; T. Li et al., 2024; Liu et al., 2025), we controlled for gender, age, and major. We controlled for student *gender* (*1 = male, 2 = female*) since prior work has found that men and women have different levels of GenAI literacy and innovation (Ji et al., 2025). Student *age* affects the various levels of critical thinking (1 = 18–22 years old, 2 = 22–26 years old, 3 = more than 26 years old). Student *major* (1 = Engineering (disciplines), 2 = Natural Sciences (disciplines), 3 = Humanities (disciplines), 4 = Medicine (disciplines), 5 = Arts (disciplines), 6 = Management (disciplines), 7 = other) indicates different kinds of innovation.

### 3.3. Reliability and Validity

Scale reliability was examined by computing Cronbach’s alpha for each construct. Every coefficient registered above the 0.70 threshold conventionally recommended for adequate internal consistency (Nunnally, 1978). To scrutinize convergent validity, a confirmatory factor analysis (CFA) was conducted. The proposed measurement model demonstrated adequate fit (χ^2^/df = 2.050, *p* < .001, CFI = 0.960, TLI = 0.951, RMSEA = 0.068, and SRMR = 0.046). As shown in Table 1, most standardized loadings reached or exceeded the 0.70 benchmark, and one item loaded slightly below this criterion. All loadings were statistically significant, which is widely regarded as a key prerequisite for establishing convergent validity (DiStefano & Hess, 2005). Moreover, the average variance extracted (AVE) for every construct satisfied the 0.50 standard (Fornell & Larcker, 1981). Taken together, the CFA evidence supports the convergent validity of the measurement instrument.

Discriminant validity was appraised via four complementary procedures. First, the heterotrait–monotrait ratio of correlations (HTMT) was calculated to gauge the degree of between-construct overlap. Table 2 reveals that all pairwise HTMT estimates stayed below the 0.85 boundary, signaling minimal redundancy across constructs. Second, following the Fornell and Larcker (1981) guideline, the square root of each construct’s AVE was compared against its correlations with the remaining latent variables; the former consistently exceeded the latter, indicating that each construct accounts for more variance in its own indicators than in those of other constructs. Third, for every focal construct, both the maximum shared variance (MSV) and the average shared variance (ASV) remained beneath the corresponding AVE value (Table 1), lending additional support for adequate discriminant validity. Fourth, nested model comparisons via CFA furnished further verification. As Table 3 indicates, the hypothesized four-factor model (χ^2^/df = 2.050, *p* < .001, CFI = 0.960, TLI = 0.951, RMSEA = 0.068, and SRMR = 0.046) outperformed the three-factor, two-factor, and one-factor alternatives in terms of overall fit, thereby reinforcing the conclusion that the constructs are empirically distinguishable.

### 3.4. Common-Method Bias

Given that the study variables were measured with self-report instruments at a single time point, the data may contain systematic variance attributable to the measurement context rather than to the substantive constructs (Podsakoff et al., 2003). Three complementary diagnostics were therefore undertaken to evaluate the potential influence of common method bias (CMB).

First, Harman’s single-factor test was applied to the complete item set. The results provided no evidence that a single latent factor captured the majority of the covariance shared by the indicators, suggesting that the correlations among the study variables are unlikely to be driven by a dominant method component. Second, a confirmatory factor analysis (CFA) contrasted the hypothesized four-factor structure with an alternative specification in which all indicators were loaded onto one factor. The unidimensional model displayed markedly poorer fit (χ^2^/df = 12.446, CFI = 0.541, TLI = 0.471, RMSEA = 0.224, SRMR = 0.180) than the four-factor model (χ^2^/df = 2.050, *p* < .001, CFI = 0.960, TLI = 0.951, RMSEA = 0.068, SRMR = 0.046), implying that a general method factor could not reproduce the observed covariance structure. Third, the unmeasured latent method construct (ULMC) technique (Ding et al., 2023) was adopted to quantify the contribution of common method variance (CMV). In this procedure, a global method factor loading on all indicators was superimposed on the four-factor model, and the augmented model was re-estimated. Relative to the baseline, the inclusion of the method factor produced no appreciable improvement in fit (χ^2^ = 225.762, df = 97, CFI = 0.950, TLI = 0.939, RMSEA = 0.076, SRMR = 0.125), with the change indices (ΔCFI = −0.01 ≤ 0.01; ΔRMSEA = 0.008 < 0.05) remaining within conventionally accepted bounds; the method factor thus absorbed only trivial incremental variance. Collectively, the three checks converge, and common method bias does not constitute a material threat to the validity of the present conclusions.

## 4. Results

To reduce the risk of collinearity between the predictors and their product terms, all independent and moderating variables were mean-centered prior to the analyses (Aiken & West, 1991). Collinearity diagnostics confirmed that the variance inflation factor (VIF) for every predictor fell between 1.020 and 2.030, comfortably below the conventional cutoff of 10. Multicollinearity, therefore, did not compromise the reliability of the regression estimates reported below.

The proposed hypotheses were tested through a series of nested ordinary least squares regressions, with predictor blocks entered incrementally (see Table 4). Because all four hypotheses were specified a priori, hypothesis tests were first evaluated at the conventional α = 0.05 level (two-tailed). As a supplementary robustness check, we also applied a Bonferroni-corrected significance level (α/4 = 0.0125; Bender & Lange, 2001) to the four hypothesis tests. Under this more stringent criterion, the coefficients for H2 (both the linear and the quadratic term) and the interaction term for H3 (*p* < .01) remained significant, whereas the interaction term for H4 did not reach even the conventional 0.05 level. In what follows, we therefore distinguish clearly between the three hypotheses that received support—H1 and H2 as direct associations and H3 as a moderating effect, with H2 and H3 additionally robust to correction for multiple comparisons—and H4, which did not receive empirical support in the present data. We accordingly refrain from claiming support for H4.

Hypothesis 1 predicted that GenAI literacy would be positively related to college students’ exploitative innovation. As Model 2 of Table 4 indicates, the coefficient for GenAI literacy was positive and statistically significant (β = 0.151, *p* < .05), lending support to H1.

**Figure 2:**
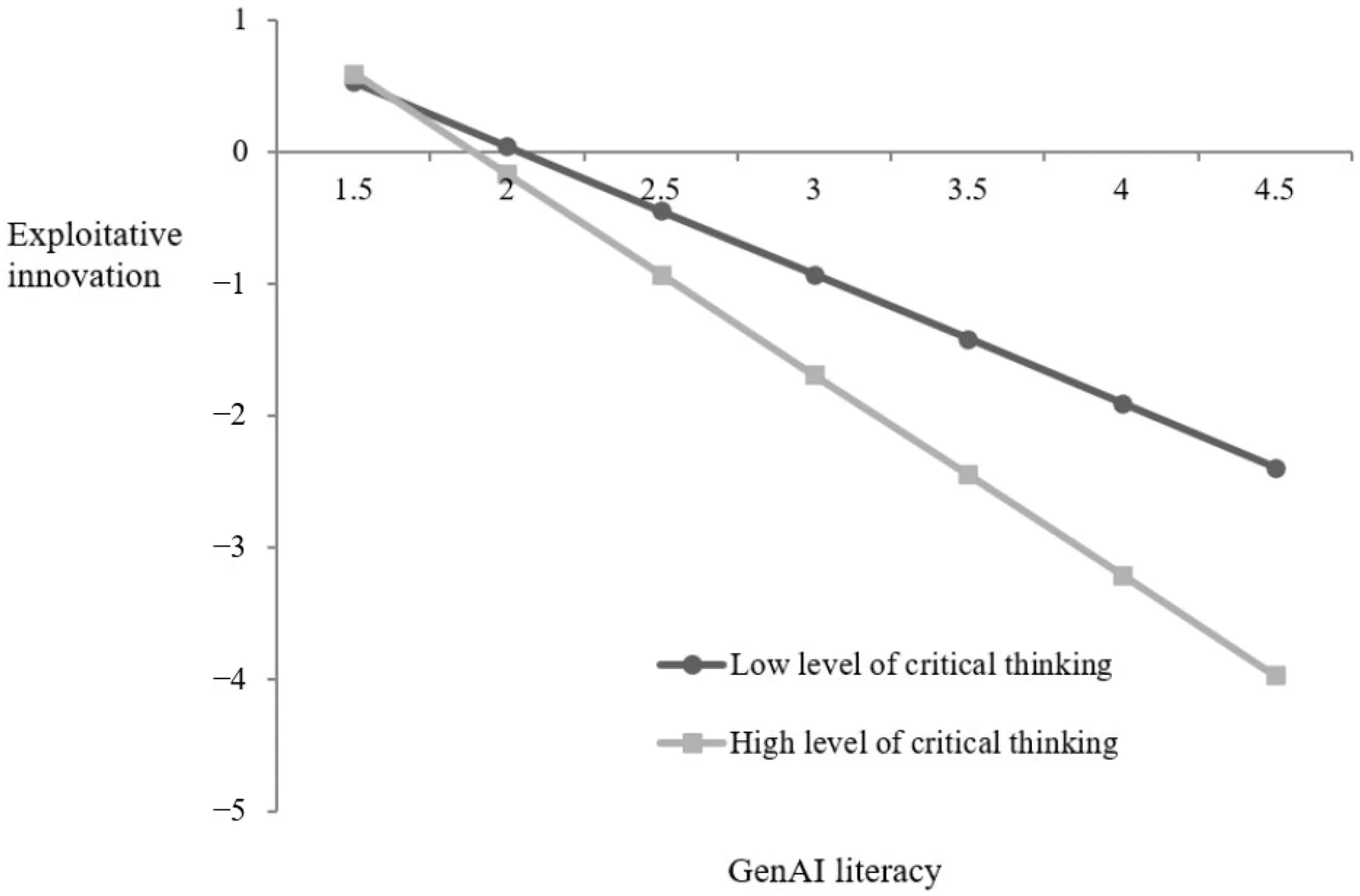
The moderating role of critical thinking on the relationship between GenAI literacy and exploitative innovation.

Hypothesis 3 stated that critical thinking would moderate the association between GenAI literacy and exploitative innovation. In Model 3, the GenAI literacy × critical thinking interaction term was negatively associated with exploitative innovation (β = −0.291, *p* < .01); this coefficient also exceeded the Bonferroni-corrected threshold (α/4 = 0.0125), and H3 was therefore supported in this study. The simple-slope pattern depicted in Figure 2 shows that the positive association between GenAI literacy and exploitative innovation appeared more pronounced among students with lower critical thinking scores, consistent with the hypothesized negative moderation.

Hypothesis 2 predicted a U-shaped association between GenAI literacy and college students’ exploratory innovation. As reported in Model 5 of Table 4, GenAI literacy showed a significant positive linear association with exploratory innovation (β = 0.486, *p* < .001), and its squared term also yielded a significant positive coefficient (β = 0.511, *p* < .01). The combination of these two coefficients is consistent with a convex (U-shaped) functional form, thereby supporting H2—an association that also remained significant under the Bonferroni-corrected criterion.

To ascertain whether this curvilinear pattern reflects a genuine U-shape rather than a simple monotonic trend, we applied the formal test developed by Lind and Mehlum (2010). This procedure evaluates three conditions: (a) the quadratic coefficient is positive and statistically significant; (b) the estimated turning point falls inside the observed range of the predictor; and (c) the slopes at the two ends of that range are significant and carry the expected signs. In our data, the turning point, computed as −β_1_/(2β_2_), equaled 4.03 on the 7-point GenAI literacy scale, which lies well within the observed interval [1, 7]. The delta-method 95% confidence interval for this point, [3.49, 4.56], was likewise contained entirely within the data range. Turning to condition (c), the slope at the lower boundary (X = 1) was significantly negative (slope = −3.09, *t* = −1.72, *p* = .042, one-tailed), implying that increments in GenAI literacy were accompanied by declines in exploratory innovation among students with limited literacy. At the upper boundary (X = 7), by contrast, the slope turned significantly positive (slope = 3.04, *t* = 2.36, *p* = .009, one-tailed), indicating that higher literacy levels were conducive to exploratory innovation. Because all three conditions were met, the evidence supports the existence of a genuine U-shaped relationship.

From a practical standpoint, these results imply that GenAI literacy begins to yield positive returns for exploratory innovation only after crossing a threshold of roughly 4.03 on the 7-point scale—a value situated slightly below the sample mean of 4.50. Below that point, the association remains negative, a pattern that may reflect cognitive overload or shallow engagement among individuals with only rudimentary familiarity with GenAI.

Hypothesis 4 proposed that critical thinking would moderate the curvilinear GenAI literacy–exploratory innovation association. In Model 6 of Table 4, the interaction between the squared GenAI literacy term and critical thinking did not reach the conventional 0.05 significance level (β = 0.179, *p* < .10). We therefore conclude that H4 did not receive empirical support in this study: given the marginal nature of the coefficient, we attach no inferential weight to it and do not interpret it as preliminary evidence or as a trend.

**Figure 3:**
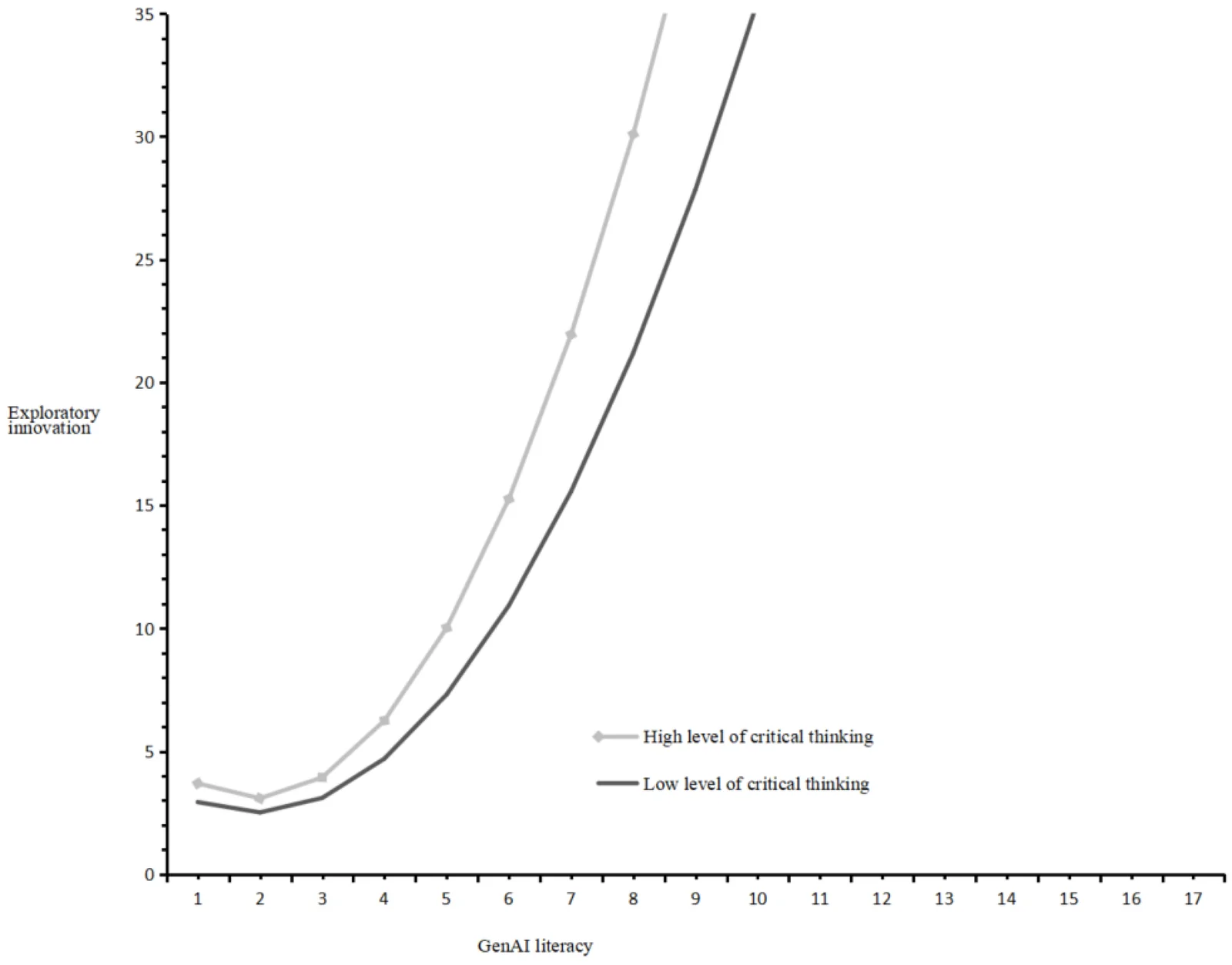
The moderating role of critical thinking on the relationship between GenAI literacy and exploratory innovation.

For descriptive completeness, Figure 3 plots the estimated curvilinear pattern at high versus low levels of critical thinking. We emphasize that this figure merely illustrates the model estimates and carries no claim of a demonstrated moderation effect. Taken together, the data indicate that the hypothesized moderating role of critical thinking in the U-shaped relationship remains an open question that future, adequately powered research should address directly.

A final caveat concerns sample selectivity. Participation was voluntary, and students who chose to complete a two-wave survey on GenAI use may have been more motivated by, and more engaged with, these tools than the broader student population. Consequently, the associations reported above may be overestimates and should be generalized only with caution.

## 5. Discussion

### 5.1. Theoretical Implications

The present study advances existing knowledge on both theoretical and empirical fronts. First, this research reveals the heterogeneous associations of GenAI literacy with distinct innovation types within educational settings. While workplace studies have explored the non-linear impact of GenAI trust on innovation (e.g., Lin et al., 2025) or documented double-edged effects on employee creativity (Guo et al., 2025), direct and differentiated tests in student learning contexts remain scarce. Specifically, unlike trust in GenAI—an attitudinal state that shows an inverted U-shaped association with exploratory innovation among employees (Lin et al., 2025)—GenAI literacy is a competence-based, learnable capability. The positive U-shaped pattern we document therefore suggests that capacity and trust may relate to innovation through distinct mechanisms: how effectively students can deploy GenAI, rather than how much they are willing to rely on it, is associated with exploratory outcomes. Moreover, students engage with GenAI under learning-oriented, formative goals rather than the performance-oriented, productivity-driven goals that characterize the workplace. The student context thus extends this theorizing to settings where self-regulated and critically evaluated use—not technology acceptance—governs whether literacy is associated with innovation. By proposing and documenting a positive linear association with exploitative innovation (H1) and a U-shaped association with exploratory innovation (H2), our study moves the educational technology literature beyond generic ‘AI enhances creativity’ narratives (e.g., Sun et al., 2025; Zhang et al., 2025), offering a precise, typology-driven framework that explains how a single competency may be linked to divergent innovation outcomes depending on the goal pursued.

Second, this study theorizes critical thinking as a key candidate moderator of the GenAI literacy–innovation linkages. While the importance of critical thinking in the AI era is widely acknowledged (e.g., G. J. Hwang et al., 2025), its specific moderating role has been neither deeply theorized nor empirically tested. Responding to concerns about over-reliance and “metacognitive laziness” (Fan et al., 2025; Zhang et al., 2025), our hypotheses (H3: negative moderation for exploitative innovation; H4: positive moderation for the U-shaped exploratory relationship) position critical thinking as a self-regulatory mechanism. The empirical picture, however, is asymmetric: the negative moderation of the GenAI literacy–exploitative innovation link received robust support (the H3 interaction was significant at *p* < .01 and survived correction for multiple comparisons), whereas the hypothesized amplification of the U-shaped exploratory relationship (H4) did not reach the 0.05 level. We therefore present the amplifying half of the dual-role account strictly as a theoretical proposition grounded in SCT’s self-regulatory mechanisms—a specification of the mechanism that generates testable predictions—whose empirical adequacy remains to be established in future research with adequately powered, preregistered designs, while treating the counteracting half (H3) as an empirically supported, though strictly associational, finding. In doing so, this study advances beyond work treating critical thinking merely as an outcome (G. J. Hwang et al., 2025) or a general concern, by articulating precisely how and why critical thinking should matter.

Third, this study advances SCT in three interrelated ways within the context of students’ GenAI innovation. (a) It deepens the mechanism of human agency by positioning critical thinking as a higher-order self-regulatory factor (Bandura, 1991) that shapes how GenAI literacy translates into heterogeneous innovation outcomes, moving beyond a focus on tool acquisition or self-efficacy—addressing concerns about “metacognitive laziness” (Fan et al., 2025). (b) It refines the triadic reciprocal determinism model (Bandura, 1986) by introducing a task-contingent view: critical thinking moderates the GenAI literacy-innovation relationships in opposite directions depending on innovation type, offering a dynamic operationalization that responds to calls for balanced GenAI use (e.g., Krakowski, 2025). (c) It reconceptualizes student–GenAI interaction as a learnable, self-regulated partnership rather than passive tool use, drawing on emerging work on reflective collaboration (Park, 2026) and deep self-regulated learning (Ji et al., 2025; G. J. Hwang et al., 2025), thereby providing a framework for modeling reflective human–AI cognitive collaboration in educational settings.

### 5.2. Practical Implications

Beyond these theoretical advances, the results yield actionable guidance for cultivating university students’ capacity for both exploitative and exploratory innovation. Specifically, they support a “Differentiated Enablement” framework for GenAI competency development—an evidence-based strategy that aligns the goals, content, and pacing of GenAI education with the specific type of innovation targeted. Because GenAI literacy relates to exploitative innovation in a linear, positive manner but to exploratory innovation in a U-shaped manner, a one-size-fits-all approach to GenAI education is suboptimal; cultivation must be differentiated by intended outcome. The framework entails three design elements.

First, goal–competency alignment: educators should specify the targeted innovation type before selecting training content. Second, pathway differentiation: for exploitative innovation (efficiency and skill optimization), the focus should be on strengthening basic GenAI skills, fostering trust, and providing task-specific training; for exploratory innovation (originality and breakthroughs), a progressive “empower first, caution later” approach is recommended—encourage broad GenAI use to expand knowledge boundaries at early stages, then shift toward preventing cognitive dependency as competency matures. Third, cognitive modulation: critical-thinking development is embedded in both pathways as a self-regulatory safeguard. We stress that the evidential basis for this third element differs across the two pathways: its counteracting function in exploitative contexts is grounded in the empirically supported H3 finding (which survived correction for multiple comparisons), whereas its amplifying function in exploratory contexts rests on theoretical reasoning rather than on a demonstrated moderating effect, given that H4 did not receive empirical support. We therefore advance the exploratory element as a theoretically grounded recommendation—one consistent with widespread concerns about over-reliance (Fan et al., 2025)—while the exploitative element is offered as an evidence-informed prescription.

This framework differs from existing approaches in three respects. Unlike generic AI literacy frameworks that enumerate competency dimensions without prescribing outcome-specific cultivation strategies, it derives training prescriptions from the empirically documented functional forms linking literacy to each innovation type. Unlike “more is better” empowerment approaches, it accommodates the U-shaped dynamics of exploratory innovation by sequencing enablement and restraint. And unlike purely restrictive “guardrail” strategies, it does not sacrifice exploratory benefits at early stages. This evidence-based framework thus provides a clear roadmap for higher education and corporate training to achieve targeted human capital development.

Second, this study highlights the potential moderating role of critical thinking and its cultivation pathways in GenAI education. We argue on theoretical grounds that critical thinking supports high-level cognitive activities such as exploratory innovation while helping to counteract overreliance on exploitative innovation, directly addressing concerns that GenAI may weaken student autonomy. We note, however, that the two halves of this argument rest on different evidential footing: the counteracting role of critical thinking in exploitative innovation received robust empirical support in our data (H3), whereas its hypothesized amplifying role in exploratory innovation (H4) did not. The following recommendations are framed accordingly—those addressing overreliance in exploitative contexts as evidence-informed, and those addressing the amplification of exploration as theory-informed propositions pending future verification. (a) Integrate critical thinking as a mandatory module in GenAI curricula—beyond technical skills, incorporate reflective dialogue, case analysis, and value judgment. (b) Design tasks with higher openness and uncertainty to push students beyond GenAI-generated “standard answers,” shifting cognitive load from information reception to deep problem-solving. Together, these recommendations support an educational paradigm that develops GenAI literacy and critical thinking synergistically, with the aim of mitigating dependency risks and cultivating future innovators for human–AI collaboration.

Third, this study provides students with a self-development guide on “How to Collaborate with GenAI to Enhance Innovation Capability.” Based on the robust threshold pattern documented in our findings, students can adopt a strategic, two-stage approach: At early stages of GenAI literacy, prioritize using GenAI to enhance learning efficiency and refine solutions (exploitative advantage). Once proficiency reaches a sufficient level, consciously leverage GenAI to inspire novel ideas and challenge existing frameworks (exploratory potential). Crucially, students should recognize that sustainable competitiveness lies not in technical proficiency alone but in critical thinking—the high-level cognitive capacity that, we argue theoretically, enables judicious use of GenAI and successful breakthrough exploration in complex, uncertain tasks. This study translates abstract theoretical models into actionable cognitive and behavioral strategies, empowering students to proactively construct their innovation capabilities in the era of human–AI collaboration.

### 5.3. Limitations and Future Research

Despite its contributions, this study has several limitations. First, the sample was modest in size (N = 228) and self-selected. Because participation was voluntary, the survey was most likely completed by students who were initially more motivated or more engaged in using GenAI; the sample therefore cannot be considered representative of the broader student population, and the reported associations may be overestimates. We have tempered the interpretation of all results accordingly and encourage readers to bear this selectivity in mind when assessing the practical significance of the findings. The modest sample size also limited statistical power, particularly for detecting interaction effects (Aguinis et al., 2005; McClelland & Judd, 1993); we accordingly interpret the non-significant H4 interaction strictly as the absence of reliable empirical support for H4—not as evidence of absence of the underlying phenomenon, and not as an effect that was “almost” detected—and we attach no inferential weight to the marginal (*p* < .10) coefficient. Second, the homogeneous student sample from a single institution limits generalizability; future research should replicate across diverse educational systems, ideally with probability sampling and with experimental or longitudinal designs that permit stronger causal inference. Third, the self-report nature of our measurements leaves the results open to common method bias; future work could mitigate this risk by drawing on behavioral analytics generated by GenAI platforms. Fourth, omitted variables remain—future research should explore additional moderators (e.g., task characteristics; Lin et al., 2025), mediators (e.g., creative self-efficacy), and double-edged effects like over-reliance. Fifth, our focus on GenAI literacy could be extended to broader AI technologies and outcomes such as problem-solving skills. In particular, future research with larger samples should directly re-test the hypothesized amplifying role of critical thinking in the exploratory (U-shaped) context and replicate its supported counteracting role in exploitative innovation, so that the remaining theoretical propositions advanced in this paper can be either established or refuted. Addressing these limitations will strengthen human–AI collaboration theory in education.

## Figures and Tables

**Table 1:** Measures and validation.

Factors and Items	Loading
GenAI literacy (GA) (AVE = 0.686, ASV = 0.107, MSV = 0.183)	
1. I actively participate in GenAI-related learning activities for digital multimodal composing	0.830 ***
2. I am dedicated to GenAI-related literacy materials to make my digital multimodal composing work comprehensible to the audience	0.885 ***
3. I am willing to spend time and effort learning how to use GenAI for better cohesion of modes in digital multimodal composing	0.828 ***
4. I use GenAI for digital multimodal composing to enhance my English/Foreign language learning performance beyond the classroom	0.855 ***
5. I will keep myself updated with the latest GenAI technologies for digital multimodal composing	0.737 ***
Critical thinking (CT) (AVE = 0.715, ASV = 0.271, MSV = 0.483)	
1. When I realize my opinion is incorrect, I am willing to change it	0.878 ***
2. During discussions with others, I strive to understand and listen to opinions different from my own	0.894 ***
3. When I encounter challenges, I try to find out the reasons and think of solutions	0.908 ***
4. When others raise questions, I reflect on matters related to their concerns	0.879 ***
5. I find that I enjoy solving challenging problems	0.639 ***
Exploitative innovation (LI) (AVE = 0.622, ASV = 0.364, MSV = 0.527)	
1. When establishing relationships, I will use my prior experience to organize and arrange activities	0.628 ***
2. When solving new problems, I will draw on the experience of others	0.831 ***
3. When organizing interactions, I will use my existing experience to organize and arrange activities	0.884 ***
Exploratory innovation (TI) (AVE = 0.651, ASV = 0.328, MSV = 0.527)	
1. I am good at proposing different ideas	0.897 ***
2. My novel ideas are often accepted by people around me	0.754 ***
3. In life, I’m good at putting forward different points of view	0.762 ***
Model Fit: χ^2^ (98) = 200.878, *p* < .001, CFI = 0.960, TLI = 0.951, RMSEA = 0.068, SRMR = 0.046	

Note: N = 228, *** *p* < .001.

**Table 2:** Descriptive statistics and correlation matrix.

Variables	1	2	3	4	5	6	7
1. Gender							
2. Age	−0.015						
3. Major	0.082	−0.051					
4. GenAI literacy	0.045	−0.091	−0.139 *	0.828	0.267	0.309	0.430
5. Critical thinking	−0.020	0.041	−0.039	0.246 **	0.846	0.743	0.526
6. Exploitative innovation	0.011	0.076	−0.100	0.268 **	0.646 **	0.789	0.526
7. Exploratory innovation	0.068	0.001	−0.070	0.379 **	0.467 **	0.577 **	0.807
Mean	1.770	3.050	4.030	4.501	5.166	5.126	4.720
SD	0.421	0.341	2.366	0.983	0.941	0.839	0.910

Note: N = 228; * *p* < .05; ** *p* < .01. The diagonal elements that are underlined are the square roots of the AVE for constructs measured with multiple items. The values above the diagonal (the upper triangle of the matrix) display the HTMT values for discriminant validity tests.

**Table 3:** Model comparison in CFA.

Model Factor	χ^2^	df	Δχ^2^	CFI	TLI	RMSEA	SRMR
Model 1: four-factor model (GA, CT, LI, TI)	200.878	98		0.960	0.951	0.068	0.046
Model 2: three-factor model (GA, CT, LI + TI)	324.525	101	123.647 (3) ***	0.914	0.898	0.099	0.064
Model 3: three-factor model (GA + CT, LI, TI)	1150.349	101	949.471 (3) ***	0.596	0.520	0.213	0.220
Model 4: three-factor model (GA, CT + LI, TI)	366.567	101	165.689 (3) ***	0.898	0.878	0.107	0.073
Model 5: two-factor model (GA + CT, LI + TI)	1258.747	103	1057.869 (5) ***	0.555	0.481	0.222	0.220
Model 6: two-factor model (GA + TI, CT + LI)	657.778	103	456.900 (5) ***	0.786	0.751	0.154	0.152
Model 7: one-factor model (GA + CT + LI + TI)	1294.435	104	1093.557 (6) ***	0.541	0.471	0.224	0.180

Note: N = 228; *** *p* < .001.

**Table 4:** Results of regression analysis.

Variables	Exploitative Innovation			Exploratory Innovation		
	Model 1	Model 2 (H1)	Model 3 (H3)	Model 4	Model 5 (H2)	Model 6 (H4)
Gender	0.023	0.010	0.010	−0.025	−0.066	−0.050
	(0.079)	(0.052)	(0.048)	(0.147)	(0.083)	(0.093)
Age	0.371 +	0.244	0.298 *	−0.265	0.080	0.084
	(0.254)	(0.193)	(0.189)	(0.290)	(0.103)	(0.110)
Major	−0.007	−0.080	−0.068	−0.076	0.013	0.017
	(0.111)	(0.059)	(0.056)	(0.138)	(0.080)	(0.073)
GenAI literacy		0.151 *	0.255 ***		0.486 ***	0.518 **
		(0.088)	(0.091)		(0.134)	(0.216)
GenAI literacy squared					0.511 **	0.478 *
					(0.256)	(0.276)
Critical thinking		0.557 ***	0.471 ***		0.443 ***	0.444 ***
		(0.081)	(0.088)		(0.106)	(0.127)
GenAI literacy × Critical thinking			−0.291 **			−0.038
			(0.124)			(0.360)
GenAI literacy squared × Critical thinking						0.179 +
						(0.128)
*F*	5.025 ***	13.903 ***	12.646 ***	3.691 **	11.492 ***	7.912 ***
*R* ^2^	0.138	0.458	0.506	0.077	0.411	0.433
Adjusted *R*^2^	0.110	0.425	0.466	0.056	0.375	0.378
*R*^2^ change		0.320 ***	0.048 **		0.334 ***	0.022

Note. N = 228. Significance levels based on two-tailed tests for all models and coefficients. + *p* < .10; * *p* < .05; ** *p* < .01; *** *p* < .001.

## Data Availability

The datasets presented in this article are not available because the data involve student participants, and the informed consent/ethics approval does not permit sharing. Therefore, requests for data access cannot be accommodated.
